# WORK TOLL: AN INTERSECTIONAL ANALYSIS ON ABSENTEEISM AND PRESENTEEISM AMONG FAMILY CAREGIVERS FOR PLWD

**DOI:** 10.1093/geroni/igae098.4259

**Published:** 2024-12-31

**Authors:** Mary Milnamow

**Affiliations:** University at Buffalo, Buffalo, New York, United States

## Abstract

Working family caregivers for PLwD experience higher rates of work productivity loss—absenteeism and presenteeism—compared to their non-caregiving counterparts. Accessible workplace supports remain out of reach for many caregivers making them vulnerable for compensation loss. Only recently has an intersectionality approach been used in quantitative research to address persistent inequalities across disciplines. This cross-sectional study examined 1) relationship between caregivers’ intersecting social identities and access to flexible hours; and 2) extent caregivers’ intersecting social identities impact experiences of absenteeism and presenteeism. Dyads (n=891) from 2017 wave of National Health and Aging Trends Study (NHATS) and National Survey of Caregivers (NSOC) were analyzed using the validated WPAI-CG measure. Mixed-effect logistic regression models and generalized linear models were conducted. Findings suggest caregivers with one full-time job were less likely to have flexible hours compared to caregivers with part-time or multiple jobs (p<.05). Black female caregivers were more likely to have flexible hours compared to Black male caregivers (OR=9.3, p=.024), but the opposite relationship was found for White caregivers (OR=0.19, p=.002). Caregivers who were female, post-HS education, with flexible hours, and multiple health conditions had greater presenteeism (p<.05). Caring for someone with dementia was associated with greater presenteeism (p<.05). Caregiving situations vary drastically. Flexible hours are popular but may not adequately address work productivity loss; furthermore, findings suggest disparate access to them. As the number of PLwD is expected to rise over the next 25 years, policies and workplaces need to consider inclusive solutions to meet the needs of working family caregivers.

